# Increased genetic diversity and prevalence of co-infection with *Trypanosoma* spp. in koalas (*Phascolarctos cinereus*) and their ticks identified using next-generation sequencing (NGS)

**DOI:** 10.1371/journal.pone.0181279

**Published:** 2017-07-13

**Authors:** Amanda D. Barbosa, Alexander W. Gofton, Andrea Paparini, Annachiara Codello, Telleasha Greay, Amber Gillett, Kristin Warren, Peter Irwin, Una Ryan

**Affiliations:** 1 School of Veterinary and Life Sciences, Murdoch University, Murdoch, Perth, Western Australia; 2 CAPES Foundation, Ministry of Education of Brazil, Brasília, Distrito Federal, Brazil; 3 Australia Zoo Wildlife Hospital, Beerwah, Queensland, Australia; 4 School of Veterinary Science, University of Queensland, Saint Lucia, Australia; University of Ostrava, CZECH REPUBLIC

## Abstract

Infections with *Trypanosoma* spp. have been associated with poor health and decreased survival of koalas (*Phascolarctos cinereus*), particularly in the presence of concurrent pathogens such as *Chlamydia* and koala retrovirus. The present study describes the application of a next-generation sequencing (NGS)-based assay to characterise the prevalence and genetic diversity of trypanosome communities in koalas and two native species of ticks (*Ixodes holocyclus* and *I*. *tasmani*) removed from koala hosts. Among 168 koalas tested, 32.2% (95% CI: 25.2–39.8%) were positive for at least one *Trypanosoma* sp. Previously described *Trypanosoma* spp. from koalas were identified, including *T*. *irwini* (32.1%, 95% CI: 25.2–39.8%), *T*. *gilletti* (25%, 95% CI: 18.7–32.3%), *T*. *copemani* (27.4%, 95% CI: 20.8–34.8%) and *T*. *vegrandis* (10.1%, 95% CI: 6.0–15.7%). *Trypanosoma noyesi* was detected for the first time in koalas, although at a low prevalence (0.6% 95% CI: 0–3.3%), and a novel species (*Trypanosoma* sp. AB-2017) was identified at a prevalence of 4.8% (95% CI: 2.1–9.2%). Mixed infections with up to five species were present in 27.4% (95% CI: 21–35%) of the koalas, which was significantly higher than the prevalence of single infections 4.8% (95% CI: 2–9%). Overall, a considerably higher proportion (79.7%) of the *Trypanosoma* sequences isolated from koala blood samples were identified as *T*. *irwini*, suggesting this is the dominant species. Co-infections involving *T*. *gilletti*, *T*. *irwini*, *T*. *copemani*, *T*. *vegrandis* and *Trypanosoma* sp. AB-2017 were also detected in ticks, with *T*. *gilletti* and *T*. *copemani* being the dominant species within the invertebrate hosts. Direct Sanger sequencing of *Trypanosoma* 18S rRNA gene amplicons was also performed and results revealed that this method was only able to identify the genotypes with greater amount of reads (according to NGS) within koala samples, which highlights the advantages of NGS in detecting mixed infections. The present study provides new insights on the natural genetic diversity of *Trypanosoma* communities infecting koalas and constitutes a benchmark for future clinical and epidemiological studies required to quantify the contribution of trypanosome infections on koala survival rates.

## Introduction

Trypanosomes are blood-borne protozoans of veterinary and medical clinical significance. In Australia, special attention has recently been given to their potential impact on health of native wildlife [1–8]. The koala (*Phascolarctos cinereus*) is an iconic Australian marsupial that is under threat of extinction across two thirds of its range, with population declines of over 50% being reported in some states [9]. Koala populations in New South Wales (NSW) and Queensland (Qld) have been listed as vulnerable under the NSW Threatened Species Conservation Act 1995 and the Qld Nature Conservation (Wildlife) Regulation 2006, respectively [10, 11]. Chlamydiosis caused by *Chlamydia pecorum* is the main disease contributing to koala population decline [12], acting in synergy with many other variables adversely affecting koala survival (such as concurrent infections with koala retrovirus-KoRV, habitat loss, vehicle collisions, climate change and dog attacks) [13, 14]. Preliminary data suggests that trypanosome infections may also be compromising the health of wild koalas, particularly those with clinical signs of concurrent diseases, thereby also contributing to population decline events [3].

Four *Trypanosoma* spp. in either single or mixed infections have been recorded in koalas to date: *Trypanosoma irwini*, *T*. *gilletti*, *T*. *copemani* and *T*. *vegrandis*, at prevalences ranging from 4.4% to 71.1% [3, 15–17]. The most common molecular method for diagnosing trypanosome infection is direct Sanger sequencing of PCR amplicons obtained using *Trypanosoma* generic primers. This method, although fast and sensitive, can impede the detection of multiple distinct co-amplified genetic variants, meaning that trypanosome co-infections can remain undetected, particularly when one trypanosome genotype is present at a lower abundance than the other [18–20]. Thus, previous studies have relied on alternative techniques such as culturing, cloning or species-specific PCR assays to resolve co-infections with trypanosomes [3, 4, 17, 21]. Such methods can, however, be relatively costly and time-consuming when screening a large amount of samples and species-specific PCR assays are limited to known species, which greatly limits the possibility of uncovering novel or rare species. On the other hand, next-generation sequencing (NGS) allows high-throughput parallelization of sequencing reactions and is therefore helpful to accurately determine the prevalence and genetic diversity of *Trypanosoma* spp. in wildlife communities and potential vectors.

Although NGS is a well-established method for profiling bacterial communities, with the exception of *Plasmodium* in mosquitoes, relatively few studies have applied this technology in the diagnostics of protozoal infections [22–25]. To the best of the authors’ knowledge, no previous published studies have used an NGS approach to investigate mixed trypanosome infections in wildlife, particularly koalas, or in potential vectors such as ticks. The development of such protocol is therefore critical, not only from a conservation awareness perspective, but also in a One Health context, as NGS has also the potential to uncover zoonotic pathogens that may be present in relatively low abundance within wildlife and tick samples.

The present study aimed to design and validate a novel NGS-based assay targeting the 18S rRNA locus to audit *Trypanosoma* communities in koala blood samples and ticks removed from koala hosts. This NGS assay was then used to determine the prevalence and genetic diversity of *Trypanosoma* sp. in koalas from eastern Australia and identify potential vectors for *Trypanosoma*.

## Materials and methods

### Sampling

A total of 168 blood samples and 91 ticks collected from koalas were investigated during the present study. Blood samples were collected during routine clinical procedures from 161 koalas (84 females and 77 males) admitted to the Australia Zoo Wildlife Hospital (AZWH), Beerwah, Qld between December 2010 and December 2011. An additional seven blood samples were collected from koalas (three females and four males) that presented to the Koala Hospital, Port Macquarie, NSW, between October 2014 and February 2015. Most of these animals originated from south-east Qld or northern NSW. For blood collection, the koalas were induced for anaesthesia with an intramuscular administration of alfaxalone (Alfaxan1 CD RTU, Jurox Australia) at a dose rate of 3 mg/kg. Anaesthesia was maintained using a combination of 1.5% isofluorane and oxygen at 1.5 L/min delivered by either mask or endotracheal intubation. Approximately 0.5–1 ml of blood was collected by venepuncture of the cephalic vein.

Of the 91 ticks collected from koalas between December 2009 to August 2014; 81 were collected at the AWZH and the remaining 10 were supplied by Endeavour Veterinary Ecology Pty Ltd, Toorbul, Qld. The ticks were stored in 70% ethanol after collection until DNA extraction. Due to differences in sampling sites, collection dates, and protocols (i.e. ticks were collected opportunistically and blood was collected during routine veterinary procedures that required anaesthesia), the ticks and blood from corresponding hosts (i.e. koala parasitized by that particular tick) were collected concurrently in only 15 cases. All aspects of sample collection were approved by the Murdoch University Animal Ethics Committee (permit numbers W2284/09 and W2636/14).

### Tick identification

Tick instars and species were microscopically examined with an Olympus SZ61 stereomicroscope (Olympus, Center Valley, PA, USA), with a Schott KL 1500 LED light source (Schott AG, Mainz, Germany), and morphologically identified using standard taxonomic keys [26, 27]. All ticks analysed in the present study belonged to the family Ixodidae; of which 56 (61.5%) were identified as *Ixodes tasmani* and 35 (38.5%) as *I*. *holocyclus*. In total, 82 (93.8%) of ticks were females. Of these, 49 (59.8%) belonged to the species *I*. *tasmani* and 33 (40.2%) to the species *I*. *holocyclus*. Only 9 ticks (7 *I*. *tasmani* and 2 *I*. *holocyclus*), were males. No nymphs or larvae were observed.

### DNA extraction

Genomic DNA was isolated from 200 μl of whole blood from koalas using a MasterPure™ DNA Purification Kit (EPICENTRE® Biotechnologies, Madison, Wisconsin, U.S.A.), according to the manufacturer’s instructions. DNA was eluted in 35 μl of TE buffer and stored at -20°C until use.

Prior to DNA isolation, ticks were surface sterilised in 10% sodium hypochlorite, washed in 80% ethanol and DNA-free PBS, and air-dried. Individual ticks were then cut into quarters with a sterile scalpel, placed in a 2 ml microtube with a 5 mm steel bead, snap frozen in liquid nitrogen for 1 min and homogenized by shaking at 40 Hz for 1 min. Genomic DNA was isolated from tick homogenates using the DNeasy Blood and Tissue Kit (Qiagen, Hilden, Germany) following the manufacturer’s recommendations. Extraction reagent blank controls were included alongside all DNA extractions. DNA extraction, PCR setup, and DNA handling procedures were all performed in separate physically contained exclusion hoods, and post-PCR procedures were performed in a separate dedicated laboratory.

### *Trypanosoma* 18S rRNA gene metabarcoding

Partial *Trypanosoma* 18S rRNA (18S) gene sequences (~350 bp) were PCR amplified from koala and tick DNA samples using a hemi-nested PCR assay utilizing the primary primers S825F [28] and TryAll R1 (5’-GACTGTAACCTCAAAGCTTTCGC-3’) (designed during the present study), and hemi-nested primers S825F and S662R [28]. Hemi-nested PCR primers also contained Illumina MiSeq adapter sequences on the 5’ end, as per standard protocols for the MiSeq platform (Illumina Demonstrated Protocols: Metagenomic Sequencing Library Preparation). PCRs were performed in 25 μl volumes containing PCR buffer, 1.5 mM MgCl_2_, 1 mM dNTPs, 0.8 μM of each primer, 0.04 U/ml Taq DNA polymerase (Fisher Biotec, Australia) and 2 μl of DNA (primary PCR).

Primary PCR products were electrophoresed through a 2% agarose gel containing SYBR Safe Gel Stain (Invitrogen, USA), visualized with a dark reader trans-illuminator (Clare Chemical Research, USA), and products corresponding to the correct length were excised, purified using a QIAquick gel extraction kit (QIAGEN, Germany). Hemi-nested PCRs used 1 μl of purified primary product as a template. Primary and hemi-nested PCRs were performed with an initial denaturation at 95°C for 3 min followed by 40 cycles of denaturation at 95°C for 30 s, annealing at 53°C (primary PCR) or 55°C (hemi-nested PCR) for 30 s, and extension at 72°C for 30 s, followed by a final extension of 72°C for 5 min. Hemi-nested PCR products were electrophoresed and purified as for the primary PCR products.

DNA samples from seven koalas (previously obtained from the Australia Zoo Wildlife Hospital) and known to harbour dual or triple trypanosome infections with *T*. *irwini*, *T*. *gilletti* and *T*. *copemani* (as determined by Sanger sequencing of cloned PCR products and/or species-specific PCR’s) [3, 16], were included in this experiment as positive controls. In addition, DNA from the pathogenic *T*. *cruzi* was included as a representative of a distantly related species from those known to infect koalas, to make sure primers were able to pick up a wide range of species including those within this important clade of trypanosomes. No-template and extraction blank controls were included in all PCR assays.

Resulting *Trypanosoma* 18S amplicons from each sample were then uniquely indexed with DNA barcodes and prepared for sequencing according to Illumina recommended protocols (Illumina Demonstrated Protocol: Metagenomic Sequencing Library Preparation), and sequenced on an Illumina MiSeq using 500-cycle V2 chemistry (250 bp paired-end reads), following the manufacturer’s recommendations.

### Bioinformatics analysis

Paired-end reads were overlapped (50 bp minimum overlap length, no mismatches allowed) and merged in USEARCH v8.0.1623 [29]. Sequences were then imported into Geneious 8.0.4 [30], where S825F and S662R primer sequences and distal bases were trimmed from the 5’ and 3’ ends of the reads. Only sequences containing the correct primer sequences (no SNPs) were retained for further analyses. In USEARCH v8.0.1623, the sequences were quality filtered (sequences with >2% expected errors were excluded from the dataset), de-multiplexed and singletons were removed on a per-run basis.

*Trypanosoma* operational taxonomic units (OTUs) were generated by clustering sequences with ≥ 99% similarity, using the UPARSE algorithm [31]. This threshold was determined based on the smallest genetic divergence at the 18S locus studied between trypanosome species found in koalas to date, according to an in-house database comprising curated *Trypanosoma* 18S sequences retrieved from GenBank. A list of species, isolate names and respective GenBank accession codes are shown in S1 Table.

After chimera filtering was carried out, the retained OTUs were BLAST-searched against the same in-house trypanosome library. Taxonomy was assigned to a species level in QIIME v.1.9.1 [32], using the BLAST algorithm (e = 0.0001). The sequences not assigned to a species level were classified as either “*Trypanosoma* sp.” (i.e. assigned to a genus-level only) or “no BLAST hits” (i.e. unassigned), and further inspected during the phylogenetic analysis.

In order to design an effective taxonomic assignment method for the present study, several algorithms and stringency parameters available in QIIME v.1.9.1 were tested, starting from highly stringent protocols (e.g. BLAST with 100% similarity threshold; BLAST with 99% similarity threshold associated with a 2/3 consensus fraction using the UCLUST algorithm). To verify the sensitivity and specificity of the algorithm selected upstream, fast phylogenetic trees were drawn in Geneious 8.0.4 (function: Fast Tree), using all assigned and unassigned OTU sequences, after each batch of test results was generated (data not shown). A number of incongruences between the NGS-BLAST taxonomic assignment and taxonomy revealed by phylogenetic analysis were observed when using more stringent parameters. For instance, many sequences classified as “unassigned” due to a lower genetic identity compared to a determined reference species (<98%), could be assigned to species according to the phylogenetic reconstructions. Therefore, the method was refined and a relatively less conservative parameter that would simply choose the BLAST “top hit” (e = 0.0001) was selected as the most reliable approach to reflect the trypanosome diversity and sequence abundance within the samples.

To exclude sampling depth heterogeneity, alpha-rarefaction plots were generated in QIIME (rarefaction was set at 14,136 and 11,158 sequence reads for koalas and ticks, respectively). The graphs suggested that a satisfactory sampling depth had been obtained to accurately represent the trypanosome community present within the samples, as curves appeared to plateau after about 2,000 reads (data not shown).

NGS data obtained during the present study is available under NCBI BioProject ID: 2588872 (Accession No. PRJNA383324).

### Phylogenetic analysis

Phylogenetic analysis was conducted on a selection of OTUs and 18S *Trypanosoma* reference sequences retrieved from GenBank. OTUs assigned to a species level that were representative of a relatively greater number of reads were selected; of which, one representative of each *Trypanosoma* sp. identified in the current study, from both koalas and ticks, were included in the phylogenetic analysis. All “unassigned” and “assigned to a genus-level only” OTUs were also added to the dataset for inspection.

Evolutionary analyses were conducted in MEGA 6 [33]. Sequences (n = 51) were aligned by CLUSTAL W [34] under the default settings. After global-trimming, the most appropriate nucleotide substitution model (i.e. with the lowest Bayesian information criterion—BIC score) was selected using the dedicated function (Find best DNA/protein model) in MEGA 6. The evolutionary history was inferred by Maximum Likelihood (ML) based on the Kimura 2-parameter model [35], using uniform rates and 95% partial deletion. The bootstrapping method (n = 500 replicates) was used to infer reliability for internal branch and the percentage (>60%) of trees, in which the associated taxa clustered together is shown next to the branches. The tree was drawn to scale, with branch lengths measured in the number of substitutions per site. All “unassigned” OTUs confirmed by phylogenetic analysis to be the result of non-specific amplifications were removed from the dataset, the analysis repeated and a final tree generated. Estimates of genetic distances between sequences were conducted using the Kimura 2-parameter model, in MEGA 6.

*Trypanosoma* 18S sequences of selected OTUs included in the phylogenetic analysis were submitted to GenBank under the following accession numbers: KX786142-KX786149 and KY640309-KY640322.

### Sanger sequencing of the 18S rRNA gene

For Sanger sequencing, the internal forward and reverse primers S825F and S662R [28] used in the hemi-nested PCR, but without the MiSeq adapters, were used in a single-round PCR assay. The resultant amplicons were separated on a 2% agarose gel, excised and purified using an in-house filter tip method [36]. The gel products were then Sanger sequenced in both directions using the ABI Prism Terminator Cycle Sequencing kit (Applied Biosystems, USA), on an Applied Biosystem 3730 DNA Analyzer. The sequences were BLAST-searched online against the GenBank nucleotide database (https://blast.ncbi.nlm.nih.gov). The results provided by Sanger and NGS methods were compared.

## Results

### Sanger sequencing analysis

In total, 59 out of 168 koala blood samples (35.1%, 95% CI: 27.9–42.8%) were positive for *Trypanosoma* DNA by single-round PCR. Sanger sequencing of amplicons obtained from these 59 samples revealed the presence of three species: *T*. *irwini*, *T*. *gilletti* and *T*. *copemani*, at prevalences of 28.6% (95% CI: 21.9–36%), 1.2% (95% CI: 0.1–4.2%) and 5.4% (95% CI: 2.5–9.9%), respectively.

A total of 23 ticks were positive by single-round PCR, however specific *Trypanosoma* DNA sequences were only obtained for 21 of these samples by Sanger sequencing (23.1%, 95% CI: 14.9–33.1%). Only two species (*T*. *gilletti* and *T*. *copemani*) were identified within ticks using Sanger sequencing, at prevalences of 17.6% (95% CI: 10.4–27%) and 5.5% (95% CI: 1.8–12.4%), respectively. No amplicons were detected after the PCR of non-template control and extraction blank control.

### NGS bioinformatics analysis

A total of 3,565,056 paired-end reads were obtained from 61 out of 175 (34.9%) koala blood samples (including the positive controls) and 23 out of 91 (25.3%) ticks. Of the total of number of sequences, 3,475,280 (97.5%) contained the correct primer sequences (no SNPs) and were retained for further analyses. During the FASTQ filtering process, 97% of the reads were retained and 3% were excluded from the dataset due to their low quality. A total of 5,990 OTUs were created during the OTU clustering step, of which 92.8% passed chimera filtering. Taxonomy was assigned to 5,562 OTUs, whilst 15 OTUs had no BLAST hits.

### Overall prevalence and molecular characterisation of *Trypanosoma* spp. by NGS

In total, 54 out of 168 koalas (32.2%, 95% CI: 25.2–39.8%) and 23 out of 91 ticks (25.3%, 95% CI: 16.7–35.5%) were positive for *Trypanosoma* spp. by NGS. The prevalence estimates of trypanosomes in *I*. *holocyclus* (34.3%, 95% CI: 19.1–52.2%) and *I*. *tasmani* (19.7%, 95% CI: 10.2–32.4%) were not significantly different (p>0.05). *Trypanosoma* infection profiling by NGS analysis revealed the presence of five *Trypanosoma* spp. (*T*. *irwini*, *T*. *gilletti*, *T*. *copemani*, *T*. *vegrandis* and *T*. *noyesi*) within koala blood samples. The same trypanosome species were detected in ticks, except for *T*. *noyesi*. Additionally, a novel species classified only as *Trypanosoma* sp. by NGS analysis was present in DNA samples from koalas, *I*. *holocyclus* and *I*. *tasmani*. All positive controls produced identical results to those obtained in previous studies which used cloning of PCR products and species-specific PCRs to identify mixed trypanosome infections [3, 16]. No trypanosome sequences were obtained from the non-template control and extraction blank controls.

**Fig 1:**
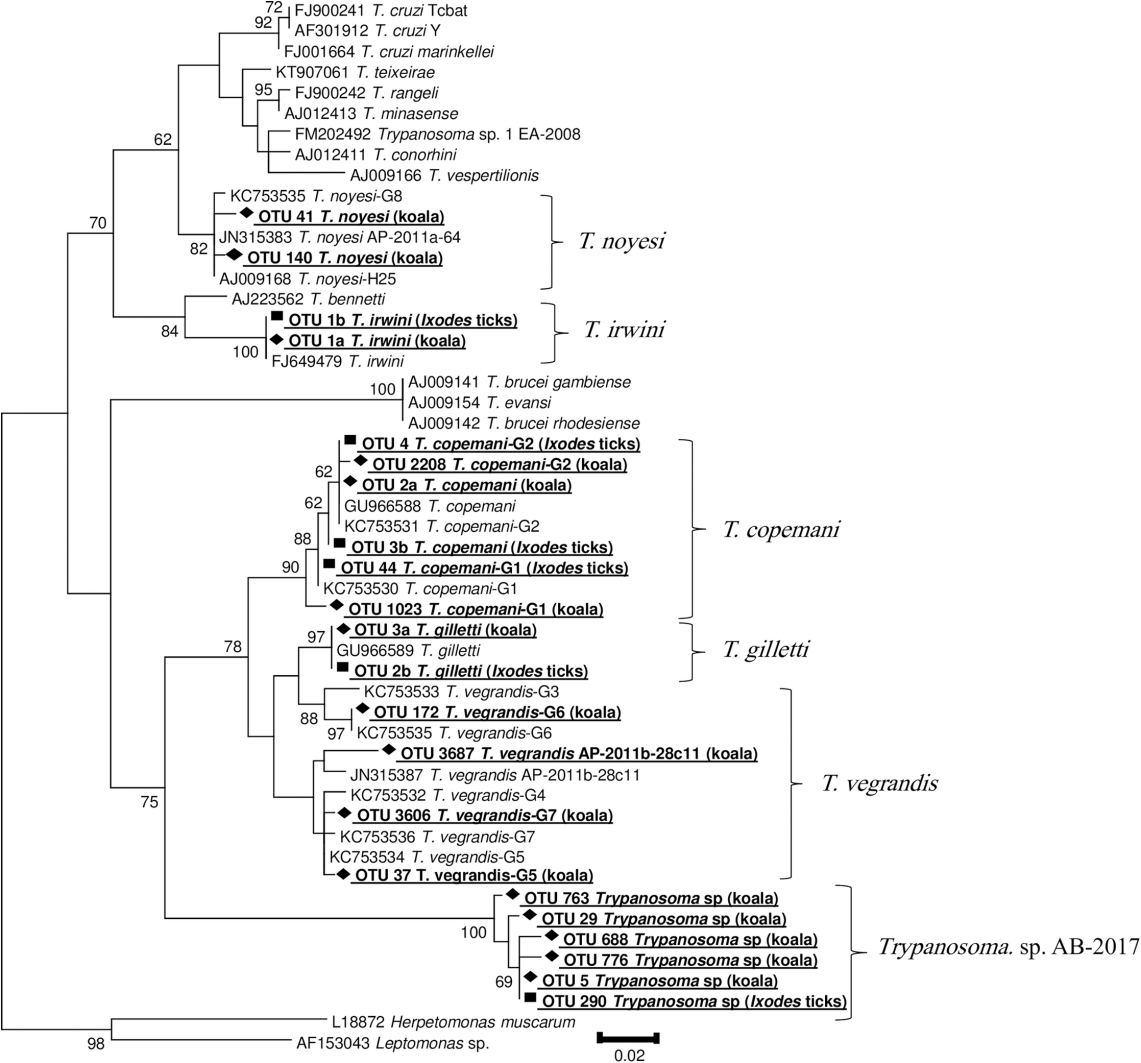
Phylogenetic analysis of a selection of NGS Operational Taxonomic Units (OTUs) assigned as *Trypanosoma* spp. and reference trypanosome sequences retrieved from GenBank. The analysis was based on 18S rDNA partial sequences (~350 bp), using the Maximum Likelihood (ML) method. Bootstrap values (>60%) are indicated at the left of each node.

Phylogenetic analysis performed on a subset of NGS OTUs, representative of a relatively greater number of sequences isolated from koalas and ticks, confirmed their molecular identity during the taxonomic assignment process (Fig 1). Phylogenetic reconstructions also revealed that the NGS method was able to discriminate distinct genotypes of *T*. *copemani* (G1 and G2) and *T*. *vegrandis* (G5-G7 and AP-2011b-28 clone 11). The pairwise identity match between OTUs and their corresponding reference sequences (i.e. species they were identified as) ranged from 97% to 100%, according to the estimates of genetic divergence matrix (data not shown). Genetic variations of up to 7% within genotypes of the same species were observed.

OTUs assigned to a genus-level only in the NGS pipeline (hereafter referred as *Trypanosoma* sp. AB-2017), clustered together and formed a separate clade strongly supported by bootstrap values (Fig 1). This clade formed a sister clade to the group consisting of *T*. *copemani*, *T*. *gilletti* and *T*. *vegrandis*. Minor genetic divergence (1–2%) was observed between the sequences within the novel clade.

### Molecular prevalence and genetic diversity of *Trypanosoma* spp. in koalas and ticks

**Fig 2:**
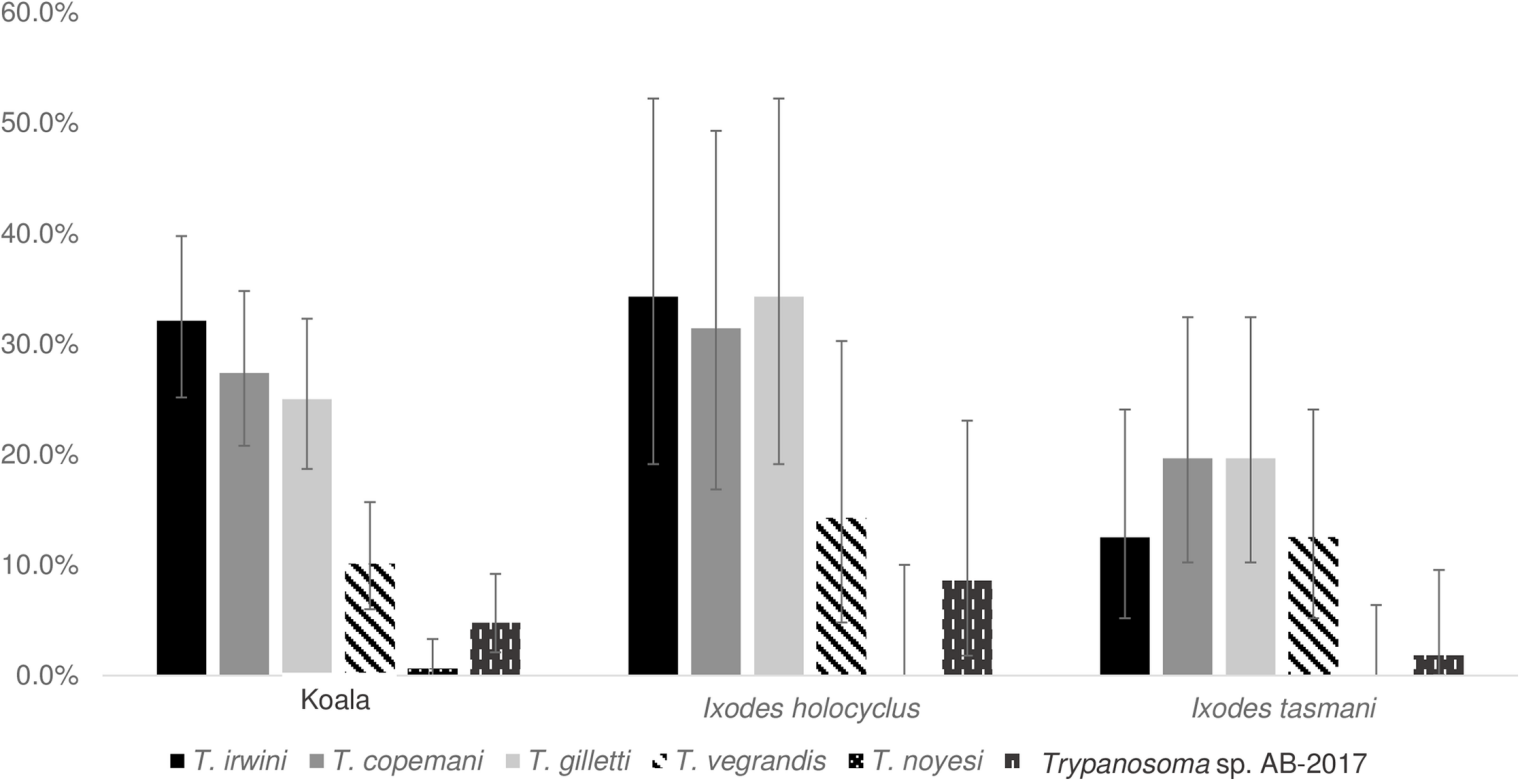
Prevalence of *Trypanosoma* spp. detected in koalas, *Ixodes holocyclus* and *Ixodes tasmani* ticks, using the NGS method. Error bars represent 95% confidence intervals (95% CI).

The prevalence of each trypanosome species detected by NGS in koalas and ticks (*I*. *tasmani* and *I*. *holocyclus*), is presented in Fig 2. In koalas, the prevalence of *T*. *irwini* (32.1%, 95% CI: 25.2–39.8%), *T*. *gilletti* (25%, 95% CI: 18.7–32.3%) and *T*. *copemani* (27.4%, 95% CI: 20.8–34.8%) was significantly higher compared to the prevalence of *T*. *vegrandis* (10.1%, 95% CI: 6.0–15.7%), *T*. *noyesi* (0.6%, 95% CI: 0–3.3%) and novel *Trypanosoma* sp. AB-2017 (4.8%, 95% CI: 2.1–9.2%). No significant difference was observed among prevalences of the three dominant species (p>0.05).

*Trypanosoma irwini*, *T*. *gilletti* and *T*. *copemani* were also the three most frequent isolates in *I*. *holocyclus*, with prevalence estimates of (34.3%, 95% CI: 19.1–52.2%), (31.4%, 95% CI: 16.9–49.3%) and (34.3%, 95% CI: 19.1–52.2%), respectively. However, the prevalence of each *Trypanosoma* spp. isolated from ticks were not statistically different from each other (p>0.05). In *I*. *tasmani* ticks, even though the prevalence estimates for *T*. *gilletti* and *T*. *copemani* (19.6%, 95% CI: 10.2–32.4%, each) were relatively higher, they were not significantly different from those of *T*. *irwini* and *T*. *vegrandis* (12.5%, 95% CI: 5.2–24.1%, each). No major differences were observed in the pattern of *Trypanosoma* spp. prevalence when comparing koalas to ticks (partially matched data) or the two tick species to each other (Fig 2).

The prevalence of each genotype of *T*. *copemani* and *T*. *vegrandis* identified in koalas and ticks was also calculated, considering the potential differences in pathogenicity and/or virulence that might exist between these distinct variants (Table 1). In koalas, the prevalence of sequences classified as *T*. *copemani* isolate Charlton was significantly higher than those of *T*. *copemani* G1 and G2 (p<0.05). As for *T*. *vegrandis*, G5 and G6 were the most common genotypes found in koalas; of which G6 showed a significantly higher prevalence compared to G3, G4, G7, AP-2011b-4c6 and AP-2011b-28c11 (p<0.05). In contrast, all genotypes of *T*. *copemani* and *T*. *vegrandis* were present in statistically similar prevalences within *I*. *holocyclus* and *I*. *tasmani* (p>0.05).

**Table 1:** Prevalence of known genotypes of *T*. *copemani* and *T*. *vegrandis* in koalas and ticks (*Ixodes holocyclus* and *Ixodes tasmani)*, determined by NGS at the 18S rRNA locus.

Trypanosome		Prevalence % (95% CI)		
Species	Genotype (GenBank accession code)	Koala	*I*. *holocyclus*	*I*. *tasmani*
*T*. *copemani*	Charlton (GU966588)	27.4 (20.8–34.8)^a^	8.2 (2.3–19.6)^a^	9.5 (4.4–17.2) ^a^
	G1 (KC753530)	1.2 (0.1–4.2)^b^	10.2 (3.4–22.2)^a,b^	8.4 (2.3–15.9) ^a,b^
	G2 (KC753531)	4.8 (2.1–9.2) ^b^	18.4 (8.8–32)^a^	9.5 (4.4–17.2) ^a^
*T*. *vegrandis*	G3 (KC753533)	0 (0–2.2) ^b^	0 (0–7.3) ^b^	0 (0–3.8) ^b^
	G4 (KC753532)	0 (0–2.2) ^b^	0 (0–7.3) ^b^	0 (0–3.8) ^b^
	G5 (KC753534)	4.2 (1.7–8.4) ^b,c^	0 (0–7.3) ^b^	0 (0–3.8) ^b^
	G6 (KC753535)	7.1 (3.7–12.1) ^c^	0 (0–7.3) ^b^	0 (0–3.8) ^b^
	G7 (KC753536)	0.6 (0–3.3) ^b^	0 (0–7.3) ^b^	0 (0–3.8) ^b^
	AP-2011b-4c6 (JN315392)	0 (0–2.2) ^b^	10.2 (3.4–22.2) ^a,b^	7.4 (3–14.6) ^a,b^
	AP-2011b-28c11 (JN315387)	0.6 (0–3.3) ^b^	0 (0–7.3) ^b^	0 (0–3.8) ^b^

Values in the same column followed by different letters are statistically distinct (p<0.05)

Among all koalas positive for *Trypanosoma* DNA by NGS (n = 54, excluding the positive controls), 34 (63%) were females and 20 (37%) were males. Overall, no significant difference between the prevalence of trypanosomes in koala females (39.1%, 95% CI: 28.8–50.1%) and males (24.7%, 95% CI: 15.8–35.5%) was observed. *Trypanosoma* DNA was only detected by NGS in female ticks; however, this result should be cautiously interpreted as the sample size of male ticks (n = 9) was considerably smaller than of females (n = 82).

### Characterization of single and mixed trypanosome infections

**Fig 3:**
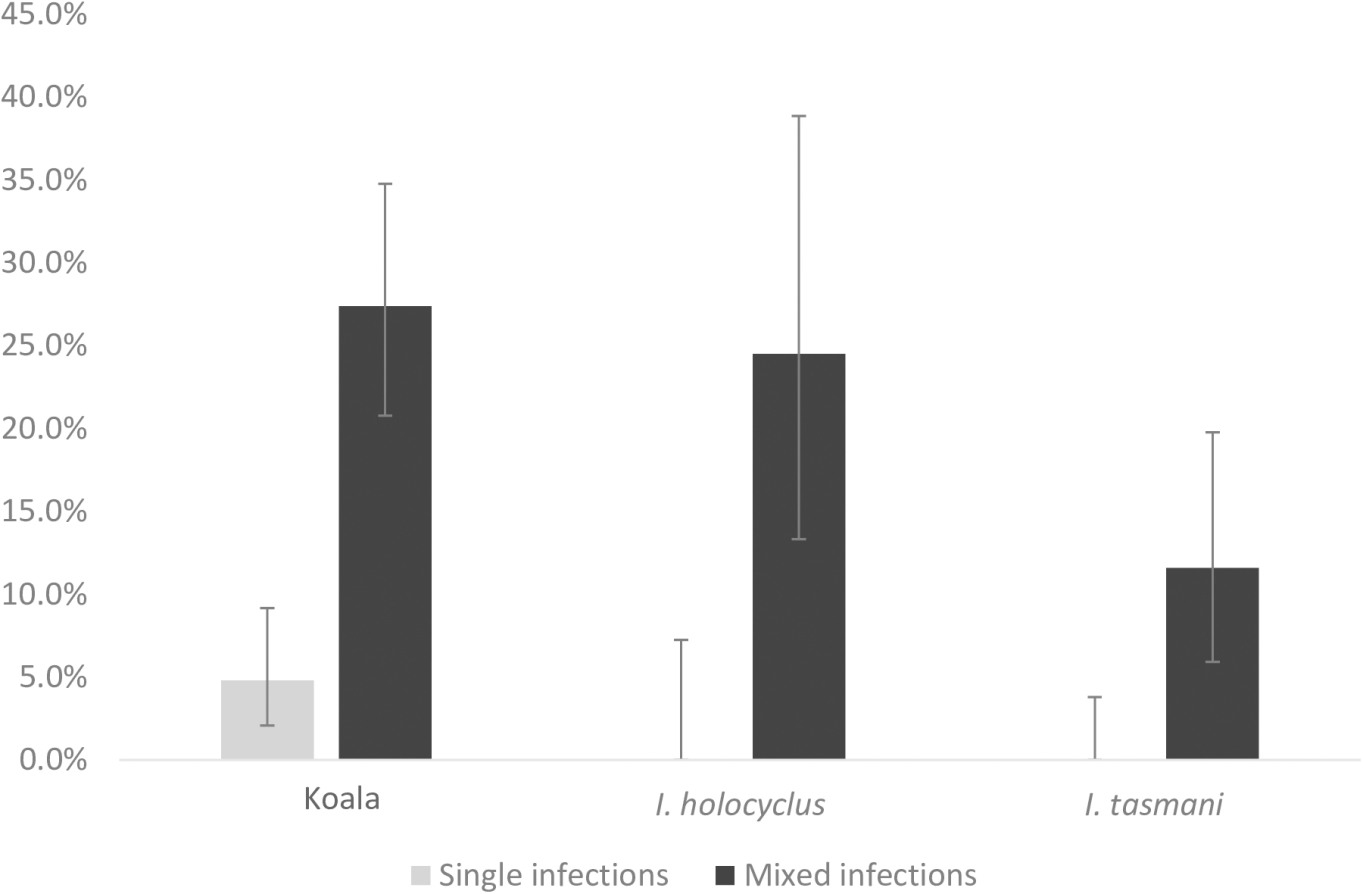
Prevalence of single and mixed trypanosome infections in koalas, *Ixodes holocyclus* and *Ixodes tasmani*, determined by NGS. Error bars represent 95% confidence intervals (95% CI).

**Fig 4:**
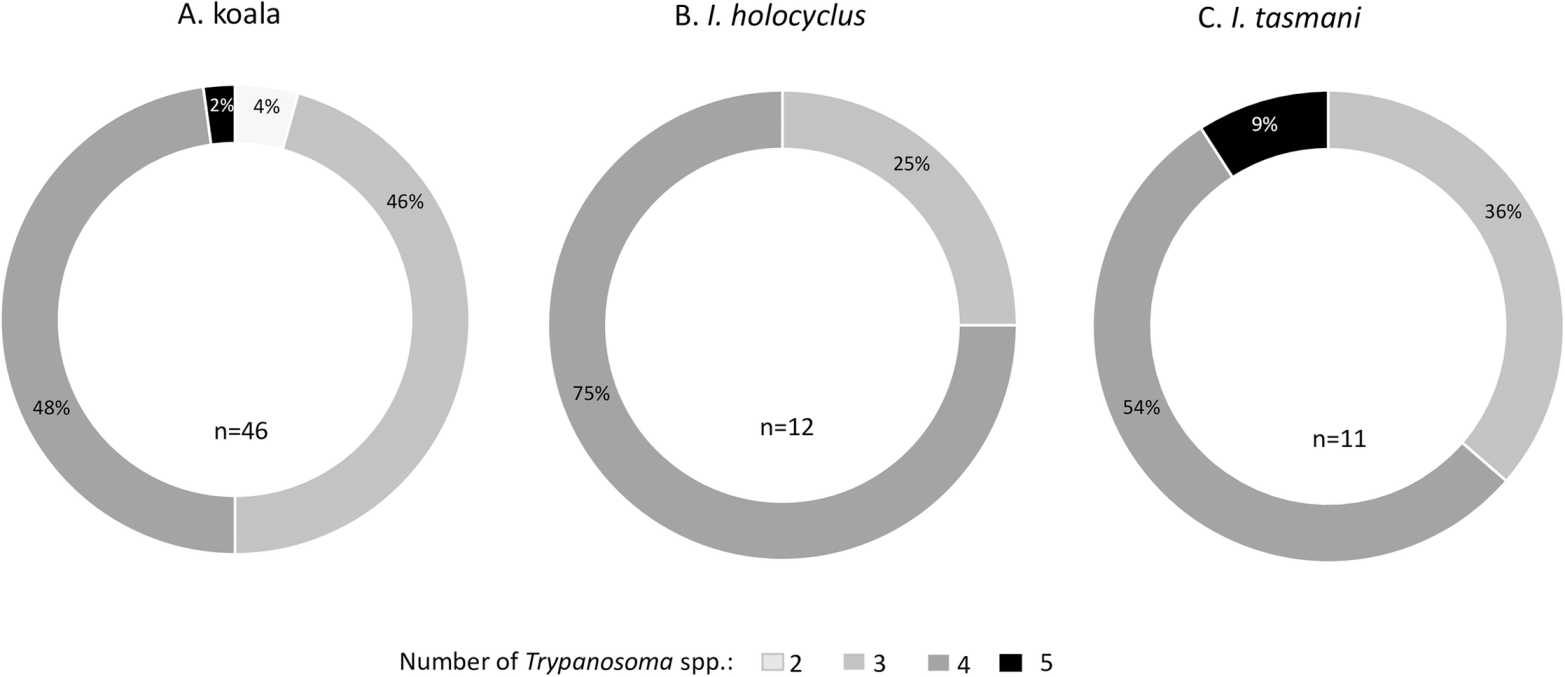
Characterisation of mixed trypanosome infections in koalas (A), *Ixodes holocyclus* (B) and *Ixodes tasmani* (C), based on the number of unique trypanosome species involved. Each doughnut chart shows proportions of the total number (n) of samples harbouring more than one *Trypanosoma* sp., based on the NGS analysis.

Intra-individual trypanosome mixed infections were observed in koalas, *I*. *holocyclus* and *I*. *tasmani*, at relatively higher prevalences compared to infections involving a single species (Fig 3). Co-infections with up to five different species were observed in koalas and ticks, not considering the genotype diversity within *T*. *copemani* and *T*. *vegrandis*. The composition of these mixed infections, in relation to the number of different species involved, is shown in Fig 4. Infections involving three and four *Trypanosoma* spp. were more frequent in koalas, whereas in ticks, the majority of samples with mixed infections, harboured four trypanosome species.

Most mixed infections with three species in koalas consisted of co-infections with *T*. *irwini*, *T*.*gilletti*, and *T*. *copemani* at a prevalence of 12.5% (95% CI: 7.9%-18.5%). Of all cases of mixed infections involving four *Trypanosoma* spp., coinfections involving *T*. *irwini*, *T*.*gilletti*, *T*. *copemani* and *T*. *vegrandis* were the most prevalent (7.7%, 95% CI: 4.2–12.8%). *Trypanosoma irwini* was the parasite identified in all cases of single infection in koalas.

In *I*. *holocyclus*, cases of multiple infections involving *T*. *irwini*, *T*.*gilletti*, *T*. *copemani* and *T*. *vegrandis* were the most prevalent (27.3%, 95% CI: 13.3%-45.5%). The majority of *I*. *tasmani* ticks that were positive for *Trypanosoma* DNA also harboured mixed infections with these four trypanosome species (prevalence: 12.2%, 95% CI: 4.6%-24.8%); however, co-infections with *T*.*gilletti*, *T*. *copemani* and *T*. *vegrandis* were relatively common among the positives (prevalence: 8.2%, 95% CI: 2.3%-19.9%).

### NGS coverage and *Trypanosoma* abundance

In total, 3,027,379 reads obtained from koala blood and ticks, using NGS, were identified as *Trypanosoma* sp. This total includes the OTUs assigned to a genus or species level and the sequences they clustered with during the OTU clustering step. Table 2 displays the total number and proportion of NGS reads assigned to each *Trypanosoma* sp. detected in koalas, *I*. *holocyclus* and *I*. *tasmani*. Overall, in koalas, a remarkably high proportion of the reads (79%) were identified as *T*. *irwini*. In contrast, over 99.8% of the sequences obtained from *I*. *holocyclus* were taxonomically assigned as *T*. *gilletti*. A high number of copies identified as *T*. *gilletti* and *T*. *copemani* were isolated from *I*. *tasmani*, which represented 50% and 46% of the total, respectively. The proportion of sequences identified as *T*. *irwini* in both tick species investigated (up to 3.1% only) was significantly lower compared to koalas (p<0.05).

**Table 2:** NGS coverage and relative abundance of taxonomically assigned sequence reads obtained from koalas and ticks (*Ixodes holocyclus* and *Ixodes tasmani*).

*Trypanosoma* spp.	Koala (n = 61*)		*I*. *holocyclus* (n = 12)		*I*. *tasmani* (n = 11)	
	Number of sequences	%	Number of sequences	%	Number of sequences	%
*T*. *irwini*	2,052,257	79.702	87	0.037	6,653	3.056
*T*. *gilletti*	254,721	9.892	234,427	99.851	109,205	50.169
*T*. *copemani*	251,770	9.778	164	0.070	100,163	46.015
*T*. *vegrandis*	9,897	0.384	72	0.031	1,649	0.758
*Trypanosoma* sp. AB-2017	6,247	0.243	26	0.011	5	0.002
*T*. *noyesi*	36	0.001	0	0	0	0
Total	2,574,928	100	234,776	100	217,675	100

*54 positive samples + 7 positive controls

The NGS data analysis revealed that *T*. *vegrandis*, *Trypanosoma* sp. AB-2017 and *T*. *noyesi* (detected in koalas only) produced a considerably lower amount of sequence reads overall compared to *T*. *irwini*, *T*. *gilletti* and *T*. *copemani*. However, analysis of sequence profile per sample revealed that these presumably rare species occurred in more than one sample, with corresponding number of reads ranging from 12 to 9,463, per sample. This provides supporting evidence that the reads were true sequences (i.e. not originated from possible sequencing errors).

To examine intra-individual genetic variation of the parasites, excluding the sampling depth heterogeneity, a composition plot was generated from rarefied data to represent the relative proportion of multiple genotypes circulating within each positive koala sample (S1 Fig) and tick (S2 Fig). The analysis shows that 100% of the koalas positive for *Trypanosoma* DNA were infected with *T*. *irwini*, and also confirms the relatively higher abundance of *T*. *irwini* sequences within most koala samples positive by NGS. The results also show a relatively high frequency of *T*. *gilletti* in ticks, which was the dominant trypanosome species within the invertebrate hosts.

### Comparison of NGS and Sanger sequencing-based assay performances

A comparison between prevalence estimates of *Trypanosoma* spp. in koalas and ticks, obtained using NGS and Sanger sequencing is presented in Table 3.

**Table 3:** Comparison of prevalence estimates of *Trypanosoma* spp. obtained using NGS and Sanger sequencing in koalas and ticks (*Ixodes holocyclus* and *Ixodes tasmani*).

Trypanosome species	Koala		*I*. *holocyclus*		*I*. *tasmani*	
	Prevalence % (95%CI)		Prevalence % (95%CI)		Prevalence % (95%CI)	
	NGS	Sanger	NGS	Sanger	NGS	Sanger
*T*. *irwini*	32.1 (25.2–39.8)	28.6 (21.9–36)	34.3 (19.1–52.2)*	0 (0–10)	12.5 (5.2–24.1)	0 (0–6.4)
*T*. *gilletti*	25 (18.7–32.3)*	1.2 (0.1–4.2)	31.4 (16.9–49.3)	28.6 (14.6–46.3)	19.6 (10.2–32.4)	12.5 (5.2–24.1)
*T*. *copemani*	27.4 (20.8–34.8)*	5.4 (2.5–9.9)	34.3 (19.1–52.2)*	0 (0–10)	19.6 (10.2–32.4)	7.1 (2–17.3)
*T*. *vegrandis*	10.1 (6.0–15.7)*	0 (0–2.2)	14.3 (4.8–30.3)	0 (0–10)	12.5 (5.2–24.1)	0 (0–6.4)
*Trypanosoma* sp. AB-2017	4.8 (2.1–9.2)	0 (0–2.2)	8.6 (1.8–23.1)	0 (0–10)	1.8 (0–6.4)	0 (0–6.4)
*T*. *noyesi*	0.6 (0–3.3)	0 (0–2.2)	0 (0–10)	0 (0–10)	0 (0–6.4)	0 (0–6.4)

*An asterisk indicates that the prevalence obtained by NGS is significantly higher than the prevalence obtained using Sanger sequencing (p<0.05), for that particular trypanosome and host species.

Although the overall prevalence of *Trypanosoma* in koalas obtained using the Sanger sequencing method (35.1%, 95% CI: 27.9–42.8%) was slightly higher than the prevalence obtained by NGS (32.2%, 95% CI: 25.2–39.8%), the specific prevalences of *T*. *gilletti* and *T*. *copemani*, were significantly underestimated by Sanger sequencing when compared to results obtained using NGS (p<0.05). Sanger sequencing also failed to identify *T*. *vegrandis*, *T*. *noyesi* and novel *Trypanosoma* sp. AB-2017 in koala blood samples.

In ticks, although the same number of positives (n = 23) was obtained by hemi-nested and single-round PCR, the overall prevalence of *Trypanosoma* sp. by NGS was slightly higher compared to that obtained using Sanger sequencing (23.1%, 95% CI: 14.9–33.1%), given that two PCR-positive samples were not confirmed to be *Trypanosoma* DNA using the latter approach. The prevalence of *T*. *copemani* in ticks estimated from Sanger sequencing results was significantly lower when compared to those obtained by NGS (p<0.05). Sanger sequencing was unable to detect *T*. *irwini*, *T*. *vegrandis* and novel *Trypanosoma* sp. AB-2017 in ticks.

In most cases of trypanosomes isolated from koala blood samples and ticks (97% and 91%, respectively), Sanger sequencing-based identifications corresponded to the most abundant species (i.e. species with greater amount of reads within the sample) identified by NGS (S2 Table and S3 Table, respectively). In addition, a total of 42 (51.2%) clean and 40 (48.8%) mixed chromatograms were obtained, which differs considerably from the proportion of single and mixed infections in both koalas and ticks, determined by NGS (Fig 3). This indicates that mixed chromatograms were not necessarily obtained in all cases of trypanosome co-infections.

### *Trypanosoma* infection profile of blood samples and ticks concurrently collected from the same koala host

In seven out of 15 cases in which tick and blood sample were concurrently collected from the same koala, both samples were negative for *Trypanosoma* DNA. In another seven cases, ticks removed from positive koalas were negative for *Trypanosoma* DNA. Interestingly, the trypanosome species characterized from a blood sample from one koala (*T*. *irwini)*, was different from that of the tick (*I*. *tasmani*) (*T*. *gilletti*) collected from the same koala, as determined by Sanger sequencing. NGS analysis identified *T*. *irwini* (17,713 reads), *T*. *copemani* (18 reads), *T*. *gilletti* (5 reads) and *T*. *noyesi* (3 reads) in the koala blood sample; whereas only *T*. *gilletti* (33,982 reads) and *T*. *copemani* (7 reads) were identified in the tick.

## Discussion

The application of NGS metabarcoding techniques can greatly improve our knowledge about the biodiversity of trypanosomes infecting Australian wildlife. This is the first study to successfully establish an NGS-based methodology, to audit trypanosome communities harboured by koalas and ticks. Notably the new method proved to be an efficient approach to characterise trypanosome polyparasitism, allowing identification of less abundant genotypes/species that are overlooked by Sanger sequencing alone.

The use of hemi-nested and not single-round PCR prior to NGS analysis constitutes a limitation of the present research, as nested PCR approaches involve an inherent risk of contamination and amplification biases [37]. However, this approach was necessary to overcome the reduction in sensitivity that the addition of MiSeq adapters to the primers had on amplification efficiency, as reported in previous studies [22, 23, 25].

To date, research using NGS platforms to investigate eukaryotic parasites have mainly focused on zoonotic pathogens [22, 24, 38–45]; hence, high-throughput methods to investigate protozoan parasitic diseases in wildlife are not yet well-established [25, 46–48]. More recently, an NGS pipeline was developed to analyse microbial eukaryotic communities, using *Eimeria* sp. as a model [25]. Although the method provides useful tools and insights, a broader use of an experiment designed specifically for *Eimeria* sp. may be problematic. This is because bioinformatics algorithms and similarity thresholds must be carefully selected and adjusted for *Trypanosoma* studies, relying on background knowledge of this parasite’s phylogeny and epidemiology. The present NGS-based assay, although performed in koala blood samples and ticks, may also prove useful in future investigations of mixed trypanosome infections in a range of other invertebrate and vertebrate hosts, including domestic animals and humans. For each case, an optimisation of PCR amplification conditions may be necessary.

The reliability of the novel methodology was assessed based on several criteria. For instance, all positive controls harbouring dual or triple mixed trypanosome infections with *T*. *irwini*, *T*. *gilletti* and *T*. *copemani*, which had previously been identified by Sanger sequencing of cloned PCR products or species-specific PCRs [3, 16], produced identical results by NGS in the present study. The assay also successfully identified the additional positive control, *T*. *cruzi*, which is relevant as surveillance of zoonotic pathogens in native species is a current research priority [49–51].

Phylogenetic analysis confirmed the classification of representative OTUs identified as *Trypanosoma* sp. in the pipeline, and demonstrated a high diversity between and within koala-derived trypanosomes. The analysis also indicated the phylogenetic position of the novel *Trypanosoma* sp. clade, which was strongly supported by bootstrap values and formed a sister clade to the group consisting of *T*. *copemani*, *T*. *gilletti* and *T*. *vegrandis*. Unfortunately, the relatively short length of amplicons, required for MiSeq sequencing, limits further inferences on the potential novel species’ genetic characterisation. Molecular analyses targeting additional loci and longer amplicons using Sanger sequencing are required for species delimitation in trypanosomes [52]. Hence, we recommend the use of NGS as the first step to investigate trypanosome genetic diversity, which should be followed by additional Sanger sequencing of longer amplicons for reliable phylogenetic characterization of any novel species identified.

NGS analysis of koala blood samples indicated that *T*. *irwini*, *T*. *copemani* and *T*. *gilletti* were the predominant trypanosome species in this marsupial, which is in line with previous findings based on Sanger sequencing [3, 16]. *Trypanosoma irwini*, besides being the most prevalent, was also the species which produced the greatest amount of sequences overall within most samples. Unfortunately, a critical challenge that limits interpretations related to NGS quantitative analyses (i.e. *Trypanosoma* abundance), is that the number of sequences generated from each variant by NGS may not reflect the number of microorganisms originally present in the sample. This is due to PCR bias (which may skew OTUs relative abundance) and to a possible variation in 18S rRNA gene copy numbers across the trypanosome species identified [53–55].

*Trypanosoma vegrandis*, *T*. *noyesi* (identified for the first time in koalas) and the novel *Trypanosoma* sp. AB-2017 OTU were found at a relatively low prevalence in koala hosts, and generated considerably less sequences compared to other co-infecting variants within koala samples such as *T*. *irwini*, *T*. *gilletti* and *T*. *copemani*. This may be the reason why these three species have not yet been detected in koalas using *Trypanosoma* sp. generic primers and Sanger sequencing. *Trypanosoma vegrandis* has only recently been isolated from koalas; and this was only possible after morphological visualization of the parasite, which led to the use of a *T*. *vegrandis*-specific assay for molecular detection [17]. However, discoveries like this may be difficult for novel species or species with low levels of parasitaemia, which is the case of *T*. *noyesi* [50].

Little is known about the clinical significance of *Trypanosoma* spp. infecting koalas. However, it is noteworthy that *T*. *gilletti* has been implicated in the decreased survival of koalas with signs of concomitant diseases; and *T*. *copemani* has been associated with anaemia in this marsupial [3]. Additionally, *T*. *copemani* has been associated with pathological changes such as muscle degeneration in woylies (*Bettongia penicillata ogilbyi*) [4, 5]. The genetic proximity of *T*. *noyesi* to the pathogenic *T*. *cruzi* is of potential concern from a conservation and public health perspective; however, intracellular stages of *T*. *noyesi*, suggestive of trypanosome pathogenicity, have not been observed in woylies [50].

Importantly, prevalences of single and mixed trypanosome infections in koalas, obtained by NGS (4.8% and 27.4% respectively), were remarkably different from those obtained by cloning associated with Sanger sequencing (51.1% and 1.5% respectively) [16]. A possible explanation is that reliance on mixed sequencing chromatograms as indicative of multiple trypanosome infections, broadly used as a selection criteria for cloning, may be inaccurate, as demonstrated by our results.

This is the first report of trypanosome polyparasitism involving up to five species. Multiple trypanosome infections involving up to 3 and 4 genotypes have been reported in koalas and woylies, respectively [3–5, 16, 17, 21]. Co-infections composed of *T*. *irwini*, *T*. *gilletti* and *T*. *copemani* were the most frequent among the koala populations sampled, followed by concomitant infections with *T*. *irwini*, *T*. *gilletti* and *T*. *copemani*. Our results differ from previous observations that co-infections involving only *T*. *irwini* and *T*. *gilletti* were predominant among koalas, but are consistent with the report of *T*. *copemani* being more common as a triple co-infection with *T*. *irwini* [3].

The implications of interactions amongst multiple trypanosomes in a single vertebrate host by either reducing or enhancing parasitaemia, virulence or pathogenicity are still unclear [51, 56, 57]. A previous molecular survey identified mixed infections involving *T*. *vegrandis*, *T*. *copemani* and *T*. *noyesi* at a higher prevalence in a declining woylie population compared to a stable population [4]. In contrast, another study suggested there may be an interspecific competition between *T*. *copemani* and *T*. *vegrandis* in woylies, whereby *T*. *vegrandis* may moderate the sequential establishment of *T*. *copemani* [5]. Further investigations are essential to elucidate whether trypanosome polyparasitism may aggravate the consequences of infection; and to quantify the contribution of trypanosome infection, in single and mixed infections, (and co-infections with *Chlamydia* and KoRV) to the koala population decline.

NGS revealed a high trypanosome genetic diversity within *I*. *holocyclus* and *I*. *tasmani*, with five species (*T*. *irwini*, *T*. *gilletti*, *T*. *copemani*, *T*. *vegrandis* and novel *Trypanosoma* sp. AB-2017) identified within these hosts. In contrast to NGS of koalas, in both tick species examined by NGS, *T*. *gilletti* was the dominant species, with the proportion of *T*. *irwini* sequences (up to 3.1%) being unexpectedly significantly lower than in koalas. However, although koalas and ticks investigated are from the same region of Australia, the fact that they constituted independent sampling groups (i.e. only a small proportion of ticks and blood samples were concurrently collected from the same koala), makes it difficult to make inferences about the host-vector-parasite relationships.

The differences in the trypanosome species identified in a koala blood sample and a tick removed from the same animal is interesting. NGS identified *T*. *irwini* in >99.8% of sequences from the koala blood sample, whereas *T*. *gilletti* was was the dominant species identified in the tick (which was also the dominant species in ticks for which a corresponding koala blood sample was not available). This suggests that the trypanosome DNA detected within the tick, was unlikely to have originated from the koala blood meal. However, it is important to note that the number of sequences assigned to each *Trypanosoma* sp. by NGS does not necessarily reflect the actual parasite numbers originally present within the sample, due to possible amplification bias and because the 18S rRNA copy numbers of the different *Trypanosome* species identified in the present study (which are currently unknown) may vary. In addition, low abundance trypanosome species in the tick may still be transmitted, particularly if they are capable of multiplying/differentiating in the vector (cyclical transmission) or if able to survive in the tick’s digestive tract until it is accidentally ingested by another host (mechanical transmission). Further research on a larger number of koala blood samples and corresponding ticks and the fate of these trypanosomes in ticks is required to better understand the role of ticks in the transmission of different trypanosome species to koalas.This is the first report of trypanosome polyparasitism in Australian native ticks, which is probably due to limitations of Sanger sequencing used in previous surveys. It is possible that, in each tick species, the most prevalent and abundant *Trypanosoma* spp. is transmitted by the tick. On the other hand, it may also be possible that ticks can individually be vectors for more than one *Trypanosoma* spp. Further research is required to clarify the vectorial role of *Ixodes* ticks in *Trypanosoma* spp. transmission.

The identification of trypanosomes in an ectoparasite does not make it a vector, as the DNA detected could represent ingested parasites from a blood meal. However, mechanical transmission where the parasite can survive in the vectors’ digestive tract and then be passed on to another host cannot be ruled out [49, 51]. Cross-transmission experiments from infected vector candidates (ticks) to uninfected mammalian host have previously been used to confirm vectors [49]. However this is often impractical and in the case of threatened marsupials such as the koala, it is unethical.

Based on parasite detection only, *I*. *australiensis* has been identified as a potential vector for *T*. *copemani* [58]; and tabanid flies (family Tabanidae) and biting midges (family Ceratopogonidae), have been suggested as candidate vectors for *T*. *noyesi* [50]. In the present study, NGS of ticks identified four koala-derived *Trypanosoma* spp. within *I*. *tasmani* and *I*. *holocyclus*. To the best of our knowledge, this is the first identification of *I*. *tasmani* as a potential vector for *Trypanosoma* spp. A previous study relying on morphological tools only has found trypanosomes in an *I*. *holocyclus* tick retrieved from a bandicoot positive for *T*. *thylacis* [59]; thus this is the first identification of *T*. *irwini*, *T*. *gilletti*, *T*. *copemani*, *and T*. *vegrandis* in *I*. *holocyclus*, which is based on molecular evidence.

The finding of *I*. *holocyclus* harbouring *T*. *copemani* could potentially be of public health significance, given the natural resistance of *T*. *copemani* to human serum [60]. Nevertheless, further research is required to determine the zoonotic potential of *T*. *copemani* and other koala-derived trypanosomes. From a One Health perspective, it is essential to monitor the presence of trypanosomes in wildlife and their ectoparasites using NGS, considering their potential to harbour zoonotic pathogens [61].

## Conclusions

In conclusion, the novel NGS-based method for the detection and characterisation of *Trypanosoma*, described in the present study, constitutes an efficient molecular tool to audit the genetic diversity of trypanosomes in koalas and candidate vectors. Our results highlight the greater accuracy of NGS compared to Sanger sequencing, as the latter clearly underestimated the prevalence of rare isolates within the samples examined; and overlooked the presence of novel species. Therefore, Sanger sequencing of PCR amplicons obtained using *Trypanosoma* generic primers is ineffective for the detection of mixed trypanosome infections in native species.

Next-generation sequencing analysis provided new insights into trypanosomes genetic diversity in koalas and identified, for the first time, two species of native ticks (*I*. *holocyclus* and *I*. *tasmani*) as vector candidates for *T*. *gilletti*, *T*. *irwini*, *T*. *copemani*, *T*. *vegrandis* and a novel koala-derived trypanosome. The discovery that mixed infections with up to five *Trypanosoma* spp. are significantly more prevalent than single trypanosome infections in koalas, constitutes a benchmark for future clinical and epidemiological studies required to quantify the contribution of trypanosomes on clinical disease in koalas, particularly in the presence of concurrent pathogens such as *Chlamydia* and KoRV. Such knowledge can support important decisions on koala health and population management, helping to stabilize population decline events.

## Supporting information

S1 Fig Composition plot illustrating the trypanosome infection profile of each positive koala sample at the 18S rRNA locus, by NGS. Rarefaction was set at 14,136 sequences.
(TIF)

S2 Fig Composition plot illustrating the trypanosome infection profile of each positive tick sample at the 18S rRNA locus, by NGS. Rarefaction was set at 11,158 sequences.
(TIF)

S1 Table GenBank accession numbers of *Trypanosoma* 18S rDNA sequences used as reference for NGS taxonomic assignment and phylogenetic analysis. (DOCX)

S2 Table Taxonomic characterisation of trypanosome-specific sequences obtained from koalas, by NGS and Sanger sequencing at the 18S rRNA locus. Values represent the number of sequences obtained by NGS, from each sample. Genbank codes correspond to the sequences’ closest matches. (DOCX)

S3 Table Taxonomic characterisation of trypanosome-specific sequences obtained from ticks, by NGS and Sanger sequencing at the 18S rRNA locus. Values represent the number of sequences obtained by NGS, from each sample. Genbank codes correspond to the sequences’ closest matches. (DOCX)
