# Supplementary figures and images for: Increased genetic diversity and prevalence of co-infection with Trypanosoma spp. in koalas (Phascolarctos cinereus) and their ticks identified using next-generation sequencing (NGS)
Source: PLoS One. 2017 Jul 13;12(7):e0181279. doi: 10.1371/journal.pone.0181279 (PMC5509321; doi:10.1371/journal.pone.0181279)

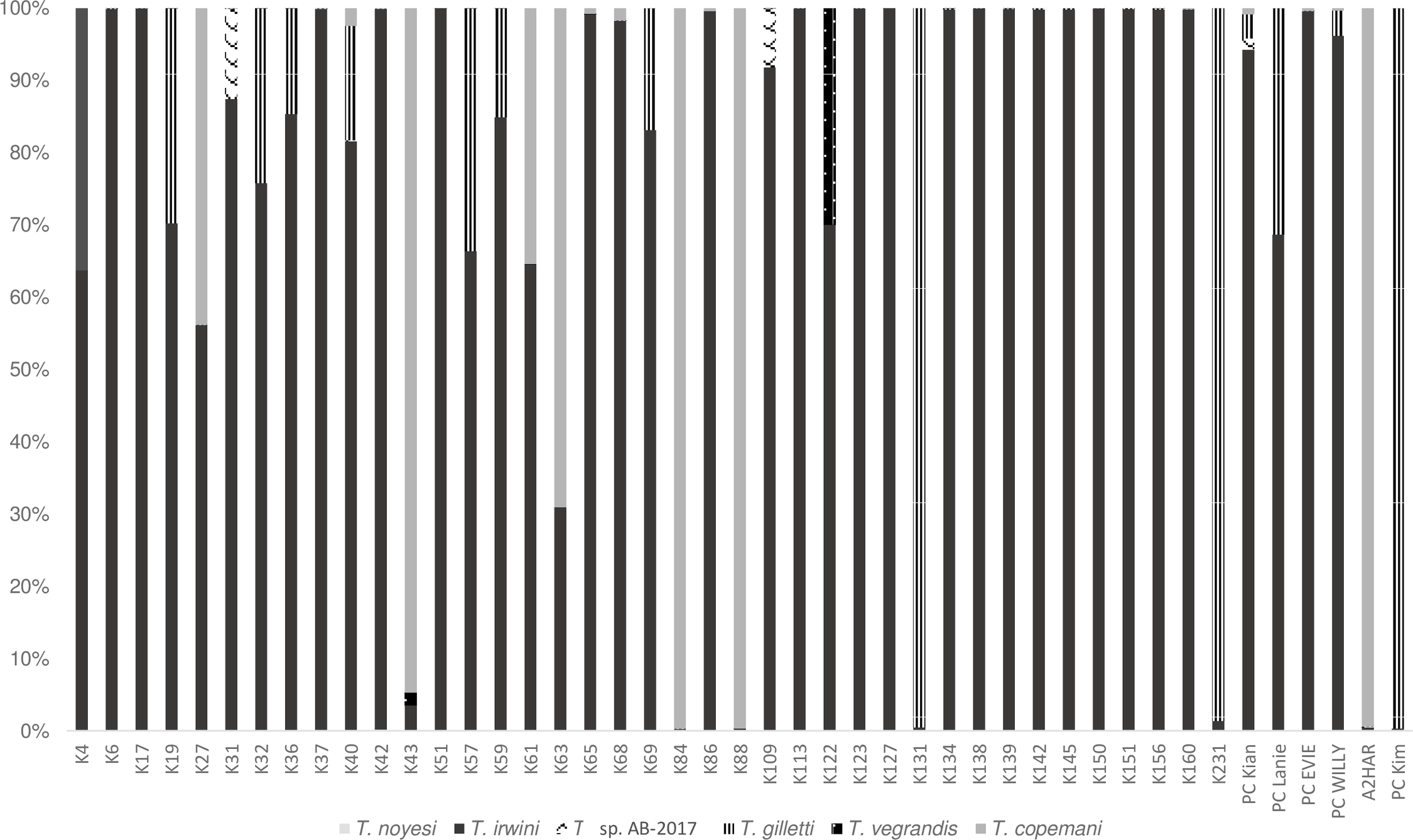

Supplement: S1 Fig — Rarefaction was set at 14,136 sequences. (TIF) [file pone.0181279.s001.tif]

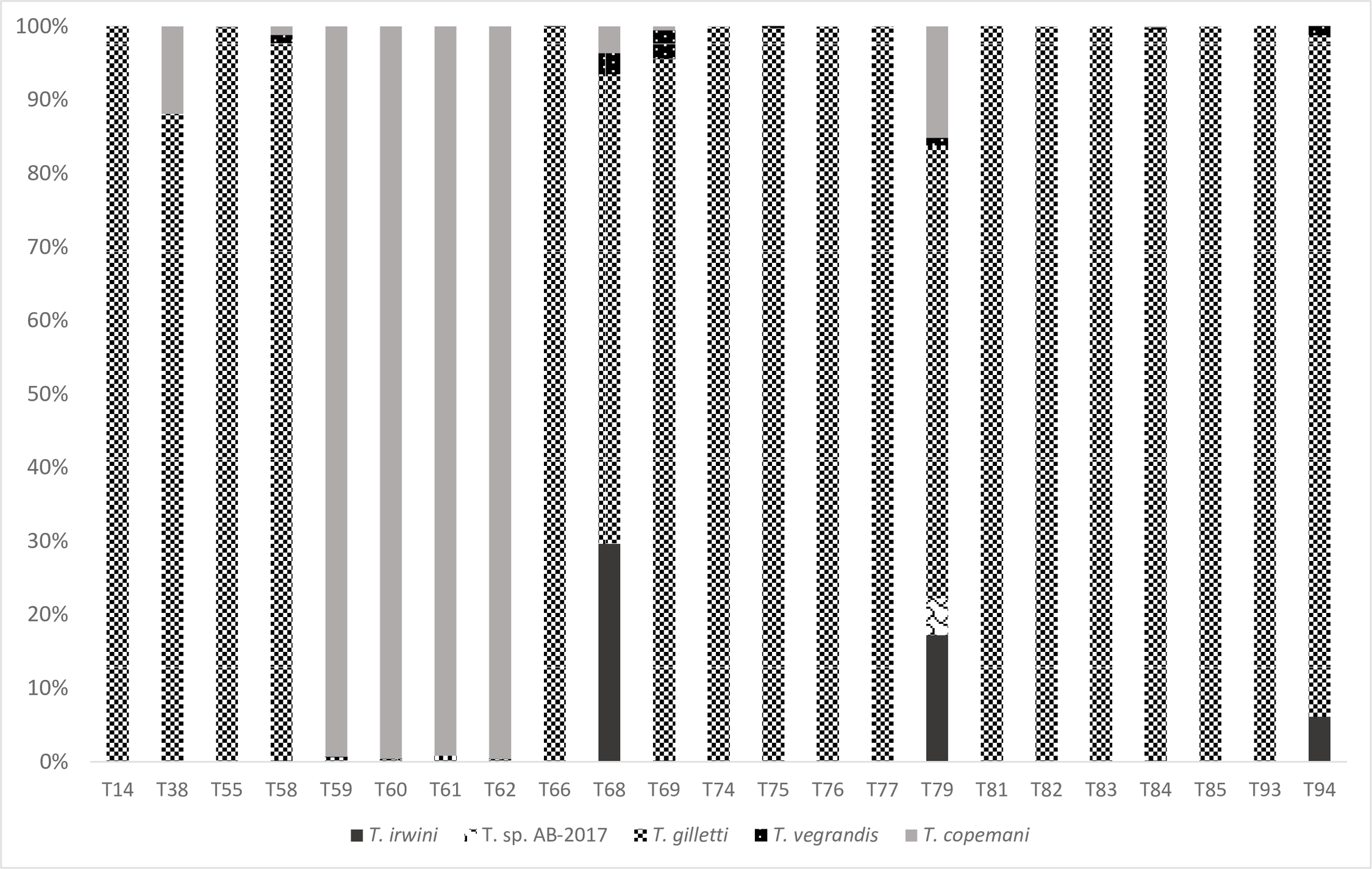

Supplement: S2 Fig — Rarefaction was set at 11,158 sequences. (TIF) [file pone.0181279.s002.tif]
