# Supplementary material for: Increased genetic diversity and prevalence of co-infection with Trypanosoma spp. in koalas (Phascolarctos cinereus) and their ticks identified using next-generation sequencing (NGS)
Source: PLoS One. 2017 Jul 13;12(7):e0181279. doi: 10.1371/journal.pone.0181279 (PMC5509321; doi:10.1371/journal.pone.0181279)
Supplement: S1 Table — (DOCX) [file pone.0181279.s003.docx]

| **Trypanosome species or isolate name** | **GenBank accession code** |
| --- | --- |
|  |  |
| *T. cruzi* Y | AF301912 |
| *T. cruzi* Tcbat | FJ900241 |
| *T. cruzi marinkellei* | FJ001664 |
| *T. cruzi* G marsupial | AF239981 |
| *T. teixeirae* | KT907061 |
| *T. rangeli* | FJ900242 |
| *T. minasense* | AJ012413 |
| *T. dionisii* | FJ001666 |
| *T. erneyi* | JN040987 |
| *T. vespertilionis* | AJ009166 |
| *Trypanosoma* 1 EA-2008 | FM202492T |
| *T. livingstonei* | KF192979 |
| *T. conorhini* | AJ012411 |
| *T. sp.* NanDoum1 | FM202492 |
| *T. sp.* HochNdi1 | FM202493 |
| *T. noyesi* H25 | AJ009168 |
| *T. noyesi* AP-2011-64 | JN315383 |
| *T. noyesi* G8 | KC753537 |
| *T. wauwau* | KT030813 |
| *T. avium* | AF416559 |
| *T. sp.* AAT | AJ620557 |
| *T. bennetti* | AJ223562 |
| *T. irwini* | FJ649479 |
| *T. lewisi* | AJ009156 |
| *T. microti* | AJ009158 |
| *T. vivax* | EU477537 |
| *T. brucei rhodesiense* | AJ009142 |
| *T. brucei gambiense* | AJ009141 |
| *T. evansi* | AJ009154 |
| *Trypanosoma sp.* ABF | AJ620564 |
| *T. copemani* Charlton | GU966588 |
| *T. copemani G1* | KC753530 |
| *T. copemani G2* | KC753531 |
| *T. gilletti* | GU966589 |
| *T. vegrandis G3* | KC753533 |
| *T. vegrandis G4* | KC753532 |
| *T. vegrandis G5* | KC753534 |
| *T. vegrandis G6* | KC753535 |
| *T. vegrandis G7* | KC753536 |
| *Trypanosoma sp.* AP-2011b 4 clone 6 | JN315392 |
| *Trypanosoma sp.* AP-2011b 28 clone 11 | JN315387 |
| *Trypanosoma sp.* AP-2011b 24 clone 4 | JN315394 |
| *T. mega* | AJ009157 |
| *T. rotatorium* | AJ009161 |
| *T. binneyi* | AJ132351 |
| *T. granulosum* | AJ620552 |
| *T.sp.* CLAR | AJ620555 |
