# Supplementary material for: Increased genetic diversity and prevalence of co-infection with Trypanosoma spp. in koalas (Phascolarctos cinereus) and their ticks identified using next-generation sequencing (NGS)
Source: PLoS One. 2017 Jul 13;12(7):e0181279. doi: 10.1371/journal.pone.0181279 (PMC5509321; doi:10.1371/journal.pone.0181279)
Supplement: S2 Table — (DOCX) [file pone.0181279.s004.docx]

| Sample ID | NGS 18S rRNA (GenBank match) | | | | | | | Sanger sequencing | Sanger sequencing (GenBank match) |
| --- | --- | --- | --- | --- | --- | --- | --- | --- | --- |
|  | *T. irwini* (FJ649479) | *T. gilletti* (GU966589) | *T. copemani* (GU966588; KC753530-32) | *T. vegrandis* ( KC753534-36; JN315387) | *T. noyesi* -H25 (AJ009168) | *T.*sp. AB-17 | Total assigned |  |  |
| K4 | 24207 | 4 | 5 | 0 | 0 | 0 | 32054 | *T. irwini* | FJ649479 |
| K6 | 25179 | 11 | 8 | 2 | 0 | 0 | 25201 | *T. irwini* | FJ649479 |
| K7 | 63 | 0 | 0 | 0 | 0 | 0 | 63 | *T. irwini* | FJ649479 |
| K12 | 167731 | 27 | 41 | 0 | 0 | 0 | 167799 | *T. irwini* | FJ649479 |
| K17 | 179247 | 168 | 21 | 0 | 0 | 0 | 179436 | *T. irwini* | FJ649479 |
| K19 | 25946 | 10978 | 6 | 1 | 0 | 1 | 36932 | *T. irwini* | FJ649479 |
| K21 | 80449 | 20 | 12 | 0 | 0 | 0 | 80481 | *T. irwini* | FJ649479 |
| K27 | 16092 | 1 | 12550 | 0 | 0 | 1 | 28644 | *T. irwini* | FJ649479 |
| K31 | 21211 | 3 | 21 | 0 | 0 | 3031 | 24266 | *T. irwini* | FJ649479 |
| K32 | 22802 | 7273 | 3 | 2 | 0 | 0 | 30080 | *T. irwini* | FJ649479 |
| K36 | 17279 | 2948 | 4 | 3 | 0 | 0 | 20234 | *T. irwini* | FJ649479 |
| K37 | 23524 | 2 | 22 | 0 | 0 | 0 | 23548 | *T. irwini* | FJ649479 |
| K39 | 140 | 0 | 0 | 0 | 0 | 0 | 140 | *T. irwini* | FJ649479 |
| K40 | 17954 | 3478 | 540 | 0 | 0 | 43 | 22015 | *T. irwini* | FJ649479 |
| K42 | 17713 | 5 | 18 | 0 | 36 | 0 | 17772 | *T. irwini* | FJ649480 |
| K43 | 801 | 24593 | 29 | 385 | 0 | 0 | 25808 | *T. copemani* | GU966588 |
| K49 | 156107 | 2 | 21258 | 0 | 0 | 0 | 177367 | *T. irwini* | FJ649479 |
| K46 | 98 | 0 | 0 | 0 | 0 | 0 | 98 | *T. irwini* | FJ649479 |
| K51 | 25650 | 5 | 6 | 1 | 0 | 0 | 25662 | *T. irwini* | FJ649479 |
| K57 | 27085 | 13707 | 10 | 1 | 0 | 0 | 40803 | *T. irwini* | FJ649479 |
| K59 | 30863 | 5462 | 7 | 2 | 0 | 0 | 36334 | *T. irwini* | FJ649479 |
| K61 | 27393 | 13 | 15031 | 1 | 0 | 0 | 42438 | *T. irwini* | FJ649479 |
| K63 | 9963 | 0 | 22243 | 0 | 0 | 0 | 32206 | *T. copemani* | GU966588 |
| K65 | 31175 | 2 | 253 | 4 | 0 | 0 | 31434 | *T. irwini* | FJ649479 |
| K68 | 23298 | 2 | 406 | 0 | 0 | 0 | 23706 | *T. irwini* | FJ649479 |
| K69 | 25943 | 5235 | 13 | 0 | 0 | 0 | 31191 | *T. irwini* | FJ649479 |
| K84 | 69 | 3 | 24541 | 0 | 0 | 0 | 24613 | *T. copemani* | GU966588 |
| K86 | 29093 | 0 | 122 | 0 | 0 | 0 | 29215 | *T. irwini* | FJ649479 |
| K88 | 97 | 4 | 29063 | 0 | 0 | 3 | 29167 | *T. copemani* | GU966588 |
| K109 | 29937 | 0 | 4 | 7 | 0 | 2658 | 32606 | *T. irwini* | FJ649479 |
| K113 | 33569 | 4 | 5 | 0 | 0 | 0 | 33578 | *T. irwini* | FJ649479 |
| K122 | 22158 | 0 | 5 | 9478 | 0 | 0 | 31641 | *T. irwini* | FJ649479 |
| K123 | 28884 | 14 | 5 | 0 | 0 | 0 | 28903 | *T. irwini* | FJ649479 |
| K127 | 21061 | 6 | 5 | 1 | 0 | 0 | 21073 | *T. irwini* | FJ649479 |
| K131 | 173 | 33357 | 7 | 0 | 0 | 0 | 33537 | *T. gilletti* | GU966589 |
| K134 | 27788 | 35 | 4 | 1 | 0 | 0 | 27828 | *T. irwini* | FJ649479 |
| K138 | 42124 | 19 | 3 | 0 | 0 | 0 | 42146 | *T. irwini* | FJ649479 |
| K139 | 30919 | 20 | 4 | 1 | 0 | 0 | 30944 | *T. irwini* | FJ649479 |
| K142 | 31747 | 41 | 15 | 0 | 0 | 1 | 31804 | *T. irwini* | FJ649479 |
| K145 | 34530 | 61 | 11 | 0 | 0 | 0 | 34602 | *T. irwini* | FJ649479 |
| K150 | 41124 | 5 | 14 | 0 | 0 | 0 | 41143 | *T. irwini* | FJ649479 |
| K151 | 27853 | 23 | 6 | 0 | 0 | 0 | 27882 | *T. irwini* | FJ649479 |
| K156 | 25530 | 35 | 9 | 3 | 0 | 0 | 25577 | *T. irwini* | FJ649479 |
| K160 | 45382 | 11 | 70 | 0 | 0 | 1 | 45464 | *T. irwini* | FJ649479 |
| K231 | 195 | 13936 | 4 | 0 | 0 | 0 | 14135 | *T. gilletti* | GU966589 |
| K237 | 302 | 0 | 0 | 0 | 0 | 0 | 302 | *T. irwini* | FJ649479 |
| K248 | 289 | 0 | 0 | 0 | 0 | 0 | 289 | *T. irwini* | FJ649479 |
| K251 | 295 | 0 | 0 | 0 | 0 | 0 | 295 | *T. irwini* | FJ649479 |
| K255 | 381 | 0 | 0 | 0 | 0 | 0 | 381 | *T. irwini* | FJ649479 |
| KH01 | 125988 | 130 | 173 | 0 | 0 | 0 | 126291 | *T. irwini* | FJ649480 |
| KH02 | 122232 | 32951 | 75 | 0 | 0 | 0 | 155258 | *T. irwini* | FJ649481 |
| KH04 | 185516 | 361 | 592 | 0 | 0 | 0 | 186469 | *T. irwini* | FJ649482 |
| KH06 | 258 | 52463 | 78916 | 0 | 0 | 0 | 131637 | *T. copemani* | GU966589 |
| KH7 | 209 | 0 | 0 | 0 | 0 | 0 | 209 | *T. irwini* | FJ649479 |
| PC Kian | 30539 | 1078 | 286 | 0 | 0 | 503 | 32406 | *T. irwini* | FJ649479 |
| PC Lanie | 35834 | 16302 | 18 | 0 | 0 | 1 | 52155 | *T. gilletti* | FJ649479 |
| PC Evie | 39510 | 3 | 148 | 0 | 0 | 0 | 39661 | *T. irwini* | FJ649479 |
| PC Willy | 40290 | 1453 | 145 | 0 | 0 | 0 | 41888 | *T. irwini* | FJ649479 |
| PC Camer | 286 | 1 | 30041 | 2 | 0 | 0 | 30330 | *T. copemani* | GU966588 |
| PC Har | 79 | 8 | 14955 | 0 | 0 | 4 | 15046 | *T. copemani* | GU966589 |
| PCKim | 103 | 28458 | 17 | 0 | 0 | 0 | 28578 | *T. gilletti* | FJ649479 |
| **Total** | 2052257 | 254721 | 251770 | 9895 | 36 | 6247 | 2568679 |  | |
