# Supplementary material for: Increased genetic diversity and prevalence of co-infection with Trypanosoma spp. in koalas (Phascolarctos cinereus) and their ticks identified using next-generation sequencing (NGS)
Source: PLoS One. 2017 Jul 13;12(7):e0181279. doi: 10.1371/journal.pone.0181279 (PMC5509321; doi:10.1371/journal.pone.0181279)
Supplement: S3 Table — (DOCX) [file pone.0181279.s005.docx]

| Sample ID |  | NGS 18S rRNA (GenBank match) | | | | | | Sanger sequencing 18S rRNA | Sanger sequencing (GenBank match) |
| --- | --- | --- | --- | --- | --- | --- | --- | --- | --- |
|  | Tick species | *T. irwini* (FJ649479) | *T. gilletti* (GU966589) | *T. copemani* (KC753530-31; GU966588) | *T. vegrandis* (JN315392) | *T*.sp. AB-17 | Total assigned |  |  |
| T14 | *I. tasmani* | 0 | 33982 | 7 | 0 | 0 | 33989 | *T. giletti* | GU966589 |
| T38 | *I. tasmani* | 0 | 13834 | 1869 | 0 | 0 | 15703 | *T. giletti* | GU966590 |
| T55 | *I. holocyclus* | 1 | 43355 | 59 | 3 | 0 | 43418 | *T. giletti* | GU966591 |
| T58 | *I. tasmani* | 4 | 2080 | 25 | 23 | 0 | 2132 | *T. giletti* | GU966592 |
| T59 | *I. tasmani* | 2 | 123 | 19945 | 5 | 0 | 20075 | *T. copemani* | KC753531 |
| T60 | *I. tasmani* | 0 | 160 | 45419 | 10 | 0 | 45589 | *T. copemani* | KC753531 |
| T61 | *I. tasmani* | 0 | 126 | 15912 | 0 | 0 | 16038 | *T. copemani* | GU966588 |
| T62 | *I. tasmani* | 7 | 48 | 16023 | 1 | 0 | 16079 | *T. copemani* | KC753531 |
| T66 | *I. holocyclus* | 10 | 23580 | 11 | 1 | 0 | 23602 | *T. giletti* | GU966589 |
| T68 | *I. tasmani* | 6577 | 14148 | 808 | 633 | 5 | 22171 | *T. giletti* | GU966590 |
| T69 | *I. tasmani* | 36 | 23762 | 136 | 948 | 0 | 24882 | *T. giletti* | GU966591 |
| T74 | *I. holocyclus* | 1 | 18114 | 14 | 0 | 0 | 18129 | *T. giletti* | GU966592 |
| T75 | *I. holocyclus* | 4 | 25063 | 0 | 57 | 9 | 25133 | *T. giletti* | GU966593 |
| T76 | *I. holocyclus* | 1 | 15255 | 5 | 0 | 0 | 15261 | *T. giletti* | GU966594 |
| T77 | *I. holocyclus* | 6 | 25586 | 9 | 10 | 0 | 25611 | *T. giletti* | GU966595 |
| T79 | *I. holocyclus* | 17 | 61 | 15 | 1 | 5 | 99 | non-specific (*Rhynchomonas nasuta*) | AY827855 |
| T81 | *I. holocyclus* | 9 | 35235 | 7 | 0 | 0 | 35251 | *T. giletti* | GU966595 |
| T82 | *I. holocyclus* | 17 | 16633 | 15 | 0 | 0 | 16665 | *T. giletti* | GU966595 |
| T83 | *I. holocyclus* | 7 | 15264 | 10 | 0 | 0 | 15281 | *T. giletti* | GU966596 |
| T84 | *I. tasmani* | 17 | 10689 | 15 | 29 | 0 | 10750 | *T. giletti* | GU966597 |
| T85 | *I. tasmani* | 10 | 10253 | 4 | 0 | 0 | 10267 | *T. giletti* | GU966598 |
| T93 | *I. holocyclus* | 9 | 16205 | 9 | 0 | 0 | 16223 | *T. giletti* | GU966599 |
| T94 | *I. holocyclus* | 5 | 76 | 10 | 0 | 12 | 103 | non-specific (*Neobodo designis*) | AY753609 |
| **Total** | | 6740 | 343632 | 100327 | 1721 | 31 | 452451 |  | |
